# Next-generation sequencing yields the first complete mitochondrial genome of the ruby dragonet *Synchiropus sycorax* (Syngnathiformes, Callionymidae)

**DOI:** 10.1080/23802359.2019.1703601

**Published:** 2019-12-18

**Authors:** Cuili Wang, Shanshan Zhou

**Affiliations:** a Key Laboratory of Environment Change and Resources Use in Beibu Gulf, Ministry of Education, Nanning Normal University, Nanning, China;; b Guangxi Key Laboratory of Earth Surface Processes and Intelligent Simulation, Nanning Normal University, Nanning, China;; c Marine Fisheries Research Institute of Zhejiang, Zhoushan, China

**Keywords:** *Synchiropus sycorax*, mitogenome, phylogenetic analysis, next-generation sequencing

## Abstract

The complete mitogenome sequence of the ruby dragonet *Synchiropus sycorax* was first determined using next-generation sequencing strategy in this study. The circle genome was 16,656 bp in length and consisted of 13 protein-coding genes, 2 ribosomal RNA genes, 22 transfer RNA genes, and 1 control region. The mitochondrial gene arrangement of *S. sycorax* is similar to those of most other fish species. Results from neighbor-joining phylogenetic analysis showed that *S. sycorax* clustered with *S. splendidus* and other species of the family Callionymidae. This study will be valuable for phylogenetic analysis of the genus *Synchiropus* and the other genera of the order Syngnathiformes.

Dragonets (family Callionymidae) are widespread and diverse in the Indo-West Pacific, Atlantic Oceans, and even Eastern Pacific (Groce et al. 2001). The ruby dragonet *Synchiropus sycorax* is a small, brilliantly Colored fish (Tea and Gill 2016) that is very popular in aquarium hobbyists. The FishBase [http://www.fishbase.org, version (08/2019)] lists 43 valid species in the genus *Synchiropus*, while only one *Synchiropus* mitogenome (*S*. *splendidus*) has been completely sequenced (Song et al. 2014). Due to the limitations of traditional morphology-based classification, more molecular cladistic studies are required to clarify genera within the family Callionymidae (Tea and Gill 2016). We hope the *S. sycorax* mitogenome could develop new DNA markers for sequence analysis, therefore facilitating species identification and phylogenetic studies.

The samples of *S. sycorax* were imported from Jolo Island, Sulu Archipelago, Philippines (6°2′N, 121°0′E) to Nanning Normal University. Total genomic DNA of *S. sycorax* was extracted from the muscle tissue, and stored at Specimen Museum of Key Laboratory of Environment Change and Resources Use in Beibu Gulf, Nanning Normal University with accession no. BGERL20191101. A ∼350-bp paired-end genomic library was prepared, and then sequenced using an Illumina Hi-Seq with 150 bp reads, generating approximately 2 Gb raw data. Three different software including SOAPdenovo2 (version 2.04) (Luo et al. 2012), SPAdes (3. 12. 0) (Bankevich et al. 2012) and Velet (1. 2. 10) (Zerbino and Birney 2008) were employed to assemble and confirm the *S. sycorax* mitogenome sequence.

The complete mitochondrial genome sequence of *S. sycorax* has been deposited in GenBank with accession no. MN732544. The circle genome (16,656 bp) comprised 13 protein-coding genes, 2 rRNA genes (12S rRNA and 16S rRNA), 22 transfer RNA (tRNA) genes and 1 control region. The nucleotide composition of the heavy strand of *S. sycorax* was 28.13% for A, 26.13% for C, 16.95% for G, and 28.79% for T, with a slight A + T bias of 56.92%. On one hand, all the protein-coding genes were initiated with ATG codon, except for the *COI*, started with GTG. On the other hand, seven protein-coding genes (*ATP6*, *COXIII*, *ND2*, *ND4*, *ND4L*, *ND5* and *ND6*) employed the typical termination codon TAA, and the remaining protein-coding genes (*ND1*, *ND3*, *COXI*, *COXII*, *ATP8* and *CYTB*) were terminated with TAG. The tRNA genes were identified by the online software tRNAScan-SE1.21 (Lowe and Eddy 1997), 22 tRNA genes were found, and the length of the 22 tRNA genes varied from 64 to 75 bp. Unlike other tRNA genes, distributed on the heavy strand, the eight tRNA genes (*tRNA^Gln^*, *tRNA^Ala^*, *tRNA^Asn^*, *tRNA^Cys^*, *tRNA^Tyr^*, *tRNA^Ser^*, *tRNA^Glu^* and *tRNA^Pro^*) were distributed on the light strand. The two ribosomal RNA genes, 12S rRNA gene (957 bp) and 16S rRNA gene (1673 bp), were located between *tRNA^Phe^* and *tRNA^Leu^* and separated by *tRNA^Val^*. The control region was located between *tRNA^Pro^* and *tRNA^Phe^*and consisted of 834 nucleotides.

A total of 12 complete mitogenome sequences from the same order Syngnathiformes have been used to construct a phylogenetic tree (Figure 1) by Neighbor-joining method (1000 bootstrap replicates, MEGA7 software) (Kumar et al. 2016). As expected, *S. sycorax* was clustered into one clade with *S*. *splendidus* and other species of the family Callionymidae. The phylogenetic tree also provided a resource for estimating the evolutionary history of the order Syngnathiformes.

**Figure 1.**
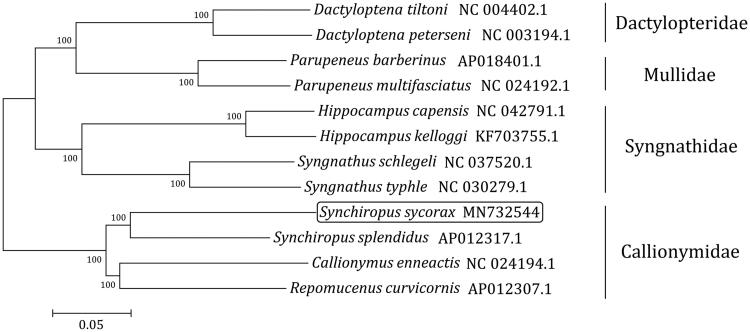
Phylogenetic analysis of 12 complete mitogenome sequences from the order Syngnathiformes using MEGA 7 by the neighbor-joining method and 1000 replications of bootstrap. The mitogenome sequence of *S. sycorax* is highlighted within a box.
